# It can't hurt to ask; a patient-centered quality of service assessment of health canada's medical cannabis policy and program

**DOI:** 10.1186/1477-7517-9-2

**Published:** 2012-01-03

**Authors:** Philippe Lucas

**Affiliations:** 1 Center for Addictions Research of BC, University of Victoria, Technology Enterprise Facility, Room 273, 2300 McKenzie Ave, Victoria, BC, V8P 5C2, Canada

**Keywords:** medical cannabis, Marihuana Medical Access Regulations, Health Canada, cannabis dispensary

## Abstract

**Background:**

In 2001 Health Canada responded to a series of Ontario court decisions by creating the Marihuana Medical Access Division (MMAD) and the Marihuana Medical Access Regulations (MMAR). Although Health Canada has conducted a small number of stakeholder consultations, the federal government has never polled federally authorized cannabis patients. This study is an attempt to learn more about patient needs, challenges and experiences with the MMAD.

**Methods:**

Launched in the spring of 2007, *Quality of Service Assessment of Health Canada's Medical Cannabis Policy and Program* pairs a 50 question online survey addressing the personal experiences of patients in the federal cannabis program with 25 semi-guided interviews. Data gathering for this study took place from April 2007 to Jan. 2008, eventually garnering survey responses from 100 federally-authorized users, which at the time represented about 5% of the patients enrolled in Health Canada's program. This paper presents the results of the survey portion of the study.

**Results:**

8% of respondents report getting their cannabis from Health Canada, while 66% grow it for themselves. >50% report that they frequent compassion clubs or dispensaries, which remain illegal and unregulated in Canada. 81% of patients would chose certified organic methods of cultivation; >90% state that not all strains are equally effective at relieving symptoms, and 97% would prefer to obtain cannabis from a source where multiple strains are available. Of the 48 patients polled that had tried the Health Canada cannabis supply, >75% rank it as either "1" or "2" on a scale of 1-10 (with "1" being "very poor", and 10 being "excellent").

**Discussion:**

72% of respondents report they are either "somewhat" or "totally unsatisfied" with Canada's medical cannabis program. These survey results and relevant court decisions suggest that the MMAR are not meeting the needs of most of the nation's medical cannabis patient community. It is hoped this research will help inform policy changes that will better address the needs of Canada's critically and chronically ill medical cannabis patient population, including the integration of community-based dispensaries into this novel healthcare delivery model.

## Background

According to the United Nations Office for Drug Control and Crime Prevention (2001) [1] cannabis is the most popular illicit substance in the world. Despite the high rate of recreational use and over 5000 years of medical use, there has never been a substantiated case of death resulting from cannabis overdose [2]. However, the therapeutic use of cannabis remains highly controversial, and only a few Western nations have introduced policies or programs to allow legal access to medical cannabis.

The Canadian government currently allows for limited access to medical cannabis through the Marihuana Medical Access Regulations (MMAR), which are administered by Health Canada's Marihuana Medical Access Division (MMAD). These court-ordered regulations are the source of much criticism by end-users and advocates, and have been found by courts to be unconstitutional in a number of decisions for unnecessarily limiting access to legal protection and a safe supply of cannabis [3-6].

Initially established in response to patient needs and ineffective or non-existent federal medical cannabis policies, community-based medical cannabis dispensaries have become the main suppliers of medical cannabis in both Canada and in many of the 14 U.S. states that have legalized the medical use of cannabis [3,7]. In Canada, community-based dispensaries, otherwise known as "compassion clubs" currently supply over 30,000 critically or chronically ill Canadians with medical cannabis [8]. Although Canadian dispensaries continue to operate without legal sanction or protection, recent research suggests that this patient-centered healthcare delivery model builds social capital and provide patients with a safe supply of cannabis within a supportive environment that's conducive to healing [3,7,5].

### A Brief History of Cannabis as a Medicine

The medical use of cannabis can be traced back at least 5000 years. The oldest reports originate in China and Egypt. It appears in a medical context in the Vedas, India's oldest religious text, and there are reports of its use as a medicine from fragments of Assyrian texts dating back to 700 B.C. The famous Chinese doctor Hua T'uo (approx. 100 A.D.) reportedly made use of a wine and cannabis mixture as an anesthetic for surgical operations [9].

There are numerous reports of the medicinal properties of cannabis from early in the nineteenth century, the most famous of which is an 1839 report titled "on the Preparations of the Indian Hemp, or Gunjah" by the Irish doctor William B. O'Shaughnessy in which he describes diverse applications for cannabis, including rheumatism, rabies, cholera, tetanus, cramps and delirium tremens [10]. A few years later Ernst Freiherr von Bibra published the renown "Narcotics and the Human Being", devoting thirty pages to the therapeutic use of cannabis preparations and hashish [11].

By the late 19^th ^Century, cannabis-based preparations were manufactured and marketed by Burroughs-Wellcome & Co. In England; and Bristol-Meyers Squib, Parke-Davis, and Eli Lilly in North America. The development of vaccines to prevent the spread of common infectious diseases, the increased use of opiates (with the introduction of the hypodermic syringe), and the discovery of aspirin at the end of the nineteenth and early twentieth century resulted in cannabis-based medicines losing their prevalence in the market place and Western pharmacopoeia [2].

In Canada, the non-medical use of cannabis was outlawed as part of the Opium and Narcotics Drugs Act of 1923, largely based on a series of misleading articles written by Emily Murphy for *MacLean's Magazine *in the early 1920's which claimed cannabis turned people into raving, blood-thirsty lunatics [12]. The US Pharmacopoeia listed Cannabis until 1941 and stated that cannabis can be used for treating fatigue, coughing, rheumatism, asthma, delirium tremens, migraine headaches, and the cramps and depressions associated with menstruation [13,2].

Although modern research into therapeutic applications for cannabis has been seriously stymied by its prohibition in most of the Western world, extensive anecdotal reports and a growing body of laboratory and clinical research suggest that it may have many medicinal uses, including hunger stimulation for wasting syndrome; anti-emetic and anti-nausea properties in AIDS or cancer chemotherapy; anti-spasmodic properties for MS, epilepsy and other neurological dysfunctions; reducing intra-ocular eye pressure in glaucoma; and analgesic properties in a large number of chronic pain conditions [14-16]. Recent research has found that cannabis can reduce the use of pharmaceutical drugs and even be an effective treatment for addiction [17-20].

### Medical Cannabis Access in Canada

Although the Canadian Addiction Survey suggests that about 1 million Canadians use cannabis for medical purposes [5], as of January 2010 the MMAD had only authorized 4884 people in Canada to use cannabis legally [21]. Additionally, the federal supply of cannabis produced by a company called Prairie Plant Systems since 2000 remains highly problematic due to a lack of strain selection, controversial production methods, and patient concerns over the quality and safety [3-5].

Problems of safe access were noted by the Canadian Senate Special Committee on Illegal Drugs in their final report on cannabis from 2002, stating that:

while a process that authorizes the possession and production of marijuana has been established in Canada, this has not ensured that cannabis is suitably available to those in need... we have come to the conclusion that the MMAR have become a barrier to access. Rather than providing a compassionate framework, the regulations unduly restrict the availability of cannabis to those who may receive health benefits from its use [22].

According to this report, one of the main reasons for the small number of applicants to the program is reluctance by physicians to act as gatekeepers to medicinal cannabis. Citing a perceived lack of information on dosage, side effects, and alternate routes of administration to smoking, both the Canadian Medical Association and the Canadian Medical Protection Agency (which insures nearly 95% of Canada's physicians) have warned against the therapeutic use of cannabis, and have recommended that doctors not participate in the federal program. For example, a CMA press release dated July 9^th^, 2003, declares:

The CMA has consistently raised concerns about the lack of evidence-based decisions to support the Medical Marijuana Access Regulations," said Dr. Dana Hanson, President of the CMA. "Our unease over use of medical marijuana has been ignored in this new policy. Physicians should not be the gatekeeper for a substance for which we do not have adequate scientific proof of safety or efficacy [23].

Such warnings have been a particular deterrent for medical specialists, whose support was initially necessary for all applicants to the program that were neither terminally ill nor likely to die in the next 12 months, such as those suffering from MS, HIV/AIDS and hepatitis C (terminal patients only required the support of a single physician). In addition, specialists were simply not available in many smaller rural communities. When compounded by the bureaucratic hurdle of filling out a 29-page application that sometimes took in excess of 12 months for Health Canada to process, the challenges to participation in this program ranged from onerous to impossible for many potential applicants.

Health Canada officially amended the MMAD application process in 2005 to remove the requirement of a supportive specialist under most circumstances. However, the new "simplified" application form was now 33 pages long, and potential applicants continue to face resistance from the medical community. The burden of this difficult application process is apparent in comparing the MMAD with the state-run Oregon Medical Marijuana Program (OMMP), one of twelve state-administered medical cannabis programs in the U.S. Although both programs originated in 1999 and have similar medical requirements for registration, Oregon's simple two page application process has led to the registration of 23,873 participants as of October 2009 (as compared to just over 4000 in Canada during the same period) - despite having a population one-tenth that of Canada [24].

### Community-Based Dispensaries

Community-based medical cannabis dispensaries, also called "compassion clubs", supply cannabis for therapeutic use upon a valid recommendation or confirmation of diagnosis from a licensed healthcare practitioner, and reflect a patient-centered response to the suffering of critically and chronically ill Canadians who might benefit from the medical use of cannabis [3-5,7].

During the late 1980's, as rates of HIV and AIDS began to rise in San Francisco, a few underground dispensaries began offering a safe source of cannabis to those needing it for medical purposes were established by compassionate people living with HIV/AIDS and drug policy reform activists. With the successful passage in 1996 of a state ballot initiative called "Proposition 215", California became the first U.S. state to allow for the legal medical use and distribution of cannabis. Within a few weeks dozens of these "compassion clubs" opened, and although they often had varied policies and practices, their common goal was facilitating access to a safe supply of cannabis for medical users [25]. Since then, over 1000 community-based medical cannabis dispensaries have opened up in California [26], and it is estimated that they currently supply over 250,000 state authorized patients [27]. Similar organizations have emerged all over the world, and in Canada and the U.S. these dispensaries remain the main source of cannabis-based medicines for therapeutic use [3].

In Canada, a loose network of community-based dispensaries provide over 30,000 critically and chronically ill Canadians access to a safe supply of cannabis within an environment conducive to healing [8]. Although Canadian dispensaries continue to operate without legal sanction or protection, communities, law enforcement, and criminal courts across Canada have shown support and tolerance for compassion clubs that self-regulate to ensure their services are strictly for medical purposes [3-5]

### Quality of Service Assessment of Health Canada's Medical Cannabis Policy and Program

Although Health Canada hosted a stakeholder consultation in 2003 to address some of the early constitutional and bureaucratic deficiencies of the MMAR, the opinion of patients registered with the MMAD has never been officially polled by the federal government in any systematic manner. This survey is an attempt to address the dearth of information about actual patient experiences with medical cannabis and Health Canada's program.

The study was funded by the McMaster Arts Research Council, and ethics approval was granted by the McMaster Research Ethics Board. Data gathering took place from April 2007 to Jan. 2008, eventually garnering survey responses from 100 federally-authorized users, which at the time represented about 5% of the patients enrolled in Health Canada's program. The 50 item self-administered survey combines multiple choice and open-ended questions, and includes items informed by validated questionnaires like the Short-Form Patient Satisfaction Questionnaire (PSQ-18) and a 2005 questionnaire designed by Belle-Isle and the Canadian AIDS Society to identify barriers to medical cannabis experienced by Canadians affected by HIV/AIDS [5,6]. In addition to basic socio-demographic data, survey questions generated by the researcher to address the history of involvement and experiences with the federal program, cannabis use patterns, and specific symptoms and conditions that cannabis has relieved.

For privacy reasons Health Canada does not make a list of federally authorized medical cannabis patients available to the public, so recruiting for this study was conducted through online and hard mail outreach to medical cannabis patient internet discussion groups and community-based dispensaries. In order to ensure that survey participants were federally authorized patients, respondents were asked to type in a specific word only found on the authorized user ID card supplied by Health Canada as a password to access the online questionnaire. Although the identity of survey respondents will be kept completely anonymous, participants were also asked to supply the registration number from their Health Canada medical cannabis ID card to allow for future verification/authentication if necessary.

### Demographic Data

Study participants were > 78% male and 20.4% female, and > 87% were 35 or older. Over 93% report that they are Caucasian, with 3 participants identifying as First Nations, 2 as Metis, and 1 as "black" (n = 97). In terms of income 36.8% make less than $20,000, and > 61% make less than $30,000, so this is a group that is well below the medium income in Canada, which may be the result of physical disabilities stemming from serious and/or chronic medical conditions. Although a medical expanse income tax claim can be filed for the cost of cannabis purchased from the government, or produced by individuals or their designated grower, there is currently no reimbursement of the actual costs of medical cannabis. In light of these findings, it is unsurprising that 46.3% of respondents state that they can "never" afford enough cannabis to relieve their symptoms. Despite the low-income levels, 77.8 had graduated from high school, and 22.3% had a university degree (Table 1). According to Statistics Canada, this is slightly higher than the Canadian average; the 2006 Census found that just over 76% of Canadians had graduated from high school, and that 18% had a university degree equivalent to a Bachelor's or higher [28].

**Table 1:** Demographics of Federally Authorized, Medical Cannabis Patients

**Female**	20.4%
**Male**	78.6%
**Caucasian**	93%
**Metis**	2%
**Black**	1%
**Other**	4%
**18-24**	2%
**25-34**	10.2%
**35-44**	23.5%
**45-54**	39.8%
**55-64**	23.4%
**65-74**	1%
**Elementary School**	5.1%
**Secondary School**	21.2%
**Technical and Non-University Education**	33.3%
**University (Undergrad, BA)**	18.2%
**University (MA, PhD, post-doc)**	5.1%
**Less than $10 000**	8.2%
**$10 000-19 999**	28.6%
**$20 000-29 000**	24.5%
**$30 000-39 000**	11.2%
**$40 000-49 000**	5.1%
**$50 000-59 000**	12.2%
**$60 000 and over**	10.2%

Although there is no way to verify that this limited sample is representative of participants in the MMAD, a recent study by Reinarman et al assessing population characteristics of 1746 California-based medical cannabis patients offers some useful comparisons. Reinarman et al found that 72.9% of their sample was male, with the researchers theorizing that the underrepresentation of women may be related to the gender-distribution of certain kinds of sports or workplace injuries, as well as the "...double stigma women face in seeking MM (medical marijuana) - for using an illicit drug and for violating gender-specific norms against illegal behavior in general" [19].

Additionally, Reinarman et al found this population to be of slightly higher education levels than the general population, with 93.1% reporting at least high school graduation, and 23.8% having a post-secondary degree, which is also similar to this Canadian survey.

### Patient Use Patterns and Preferences

**Figure 1:**
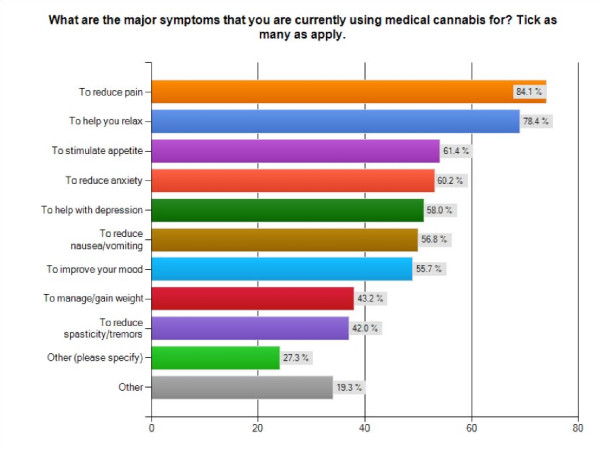
**Major Symptoms**. Bar graph of self-reported major symptoms treated with cannabis by survey participants (n = 88).

While the overwhelming majority of participants reported using cannabis recreationally prior to their medical use, > 20% were cannabis-naïve prior to using it medically (n = 89). The average years of medical use is just over 10 years, which may be reflective of the older patient profile and additionally suggests that many patients have been using cannabis for far longer than Health Canada's federal program has been in existence. When asked to check off all the major symptoms for which they used medical cannabis, most cited multiple symptoms: 84.1% cited pain relief, 78.4% cited relaxation, 61.4% cited appetite stimulation, 60.2% cited anxiety reduction, 58% cited depression, 56.8% cited nausea reduction/vomiting, 55.7% cited mood improvement, 43.2% cited desire to manage/gain weight, 42% cited reduction in spasticity/tremors, and 23.9% cited side-effects of other medications. Of interest is the high number of individuals using cannabis for relaxation, anxiety reduction, depression and mood improvement, suggesting that patients with physical health conditions may also be self-medicating for mental health issues and/or general improvements in their quality of life (Figure 1).

**Figure 2:**
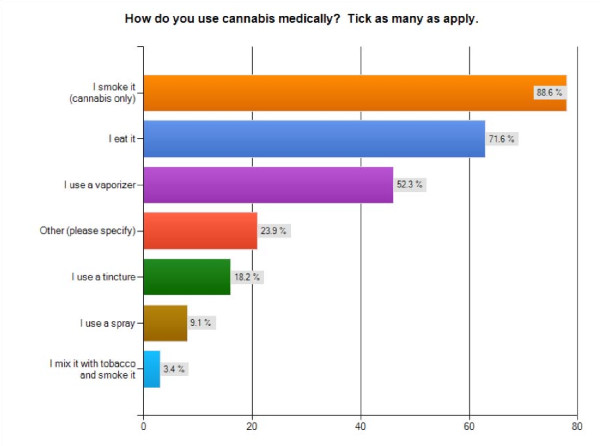
**Methods of Ingestion**. Bar graph of self-reported methods of cannabis ingestion reported by survey participants (n = 88).

In terms of personal use patterns, over 94% stated that they use it every day, which is considerably higher than the 67% reported by Reinarman et al from their California patient survey [19]. Over 88% smoke cannabis, and 71.6% report that they eat it. Over 52% have used vaporizers, 18.2% use tinctures and, unlike Europe, less than 4% mix it with tobacco. While the rate of smoking is similar to the Reinarman et al sample, which found that 86.1% smoke cannabis [19], the comparatively higher use oral ingestion/edibles (71.6% v. 24.4%) and vaporizers (52% v. 21.8%) in the Canadian sample may suggest a greater level of concern and mitigation for potential health impacts associated with smoking within the Canadian patient population [29] (Figure 2). This health awareness may also explain why 80.7% of respondents prefer to use cannabis grown using certified organic cultivation methods, whereas 19.3% either don't care (14.5%) or prefer non-organic cultivation (4.8%).

In terms of patient preferences and treatment efficacy, 90.9% report that not all strains are equally effective at relieving their symptoms. As a result, 97.6% would prefer to obtain cannabis from a source that offers a "large selection of different strains" rather than 1 or 2 strains, and over 90% would prefer to have access to raw cannabis as well as other methods of ingestion like baked goods, tinctures, and hashish, compared with 9.8% who would prefer a cannabis-only outlet. This creates a stark contrast between access through Health Canada and through community-based dispensaries. While Health Canada offers a single strain of raw cannabis and no alternatives to smoking, dispensaries make multiple strains and methods of ingestion other than smoking available to patients, including edibles, oils, tinctures, salves, and even oromucosal sprays [3].

When asked about other cannabinoid-based pharmaceutical medicines like Marinol (dronabinol), Cesamet (nabilone) and Sativex, 34.9% had tried Cesamet, 33.7% had tried Marinol, and 14% had tried Sativex. 43% had not tried any of the above, and 81.5% stated that didn't use any of these pharmaceuticals on a regular basis.

### Patient Access to Medical Cannabis

**Figure 3:**
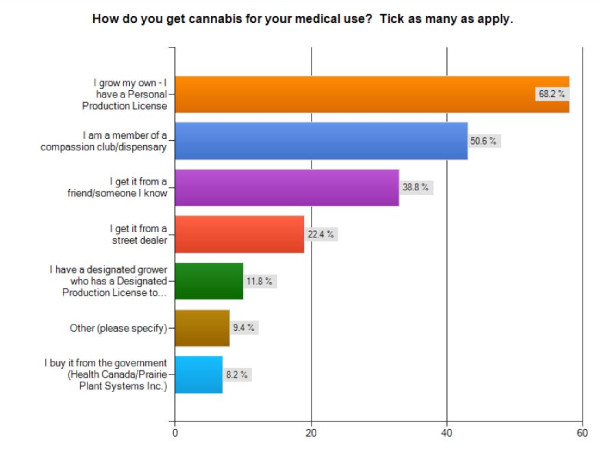
**Access to Cannabis**. Bar graph showing how survey participants access medical cannabis (n = 85).

When asked how they obtain cannabis, only 8.2% of respondents report getting their cannabis from Health Canada (although nearly half state that they have tried the federal supply), while 80% grow it for themselves or have it grown for them by a Designated Producer. Over 50% report that they frequent compassion clubs or dispensaries, 38.8% report getting it from a friend, and > 22% get their medicine from street dealers (Figure 3).

**Figure 4:**
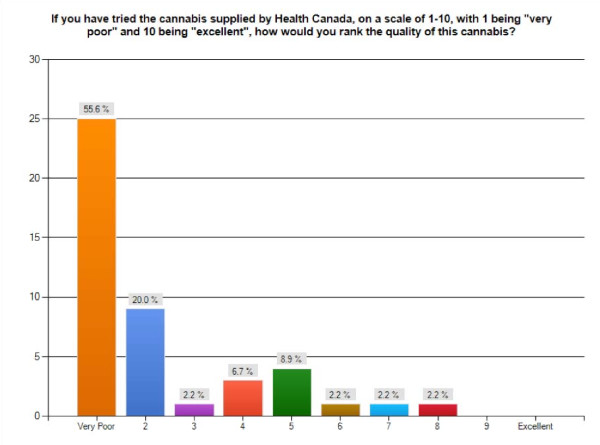
**Assessing Health Canada's Cannabis Supply**. Bar graph showing levels of satisfaction with the Health Canada medical cannabis supply on a scale of 1-10, with 1 being "very poor" and 10 being "excellent" (n = 45).

When asked how they would rank the quality of the cannabis from their regular source, 87.8% rank it as 7 or above in a scale of 1-10, with 1 being "Very Poor", and 10 being "Excellent". By comparison, of the 41 patients who have tried the federal cannabis supply, over 75% rank it as either 1 or 2 on a scale of 1-10. While 3 respondents ranked it as either a 6, 7, or 8, no one ranked it any higher (Figure 4).

Since Health Canada's cannabis supply went through some modest improvements in regards to the size of the grind, humidity level, and amount of THC in August 2004, respondents were asked when they tried this cannabis. Of the 39 who answered this question, 37 (or > 94%) used the federal supply between 2005-2007, and 2 used it before that. As such, it can be deducted that the general dissatisfaction with the quality of the federal cannabis supply is based on patient experiences with the most recent "improved" version of this product.

When asked what their single preferred source for medical cannabis would be, 65.1% stated that they would like to grown their own, 24.1% cited dispensaries, 6% would like to get their medicine from a pharmacy, 4.8% would like to get it from a friend, while neither street dealers nor Health Canada were cited by a single patient as their preferred source. This is highly relevant since Health Canada's proposed regulatory changes include removing the right for individuals to produce their own cannabis, despite this being the preferred option cited by most study participants and the option chosen by the majority of patients in the federal program [30]. As of January 2010 (the latest statistics available on the Health Canada website) 3576 out of 4884 - or over 73% - of federally authorized patients chose to produce their own medicine or to have a Designated Producer do so for them [21]. If Health Canada intends to make this program more patient-centered, removing the right for patients to produce their own supply does not appear to reflect current patient needs, and as such this proposed significant amendment to the program should be highly controversial, and will likely lead to further court challenges by patients wishing to control the cost and quality of their supply of medicine.

### Patient Experiences With Health Canada Marihuana Medical Access Division

Of study participants, nearly half (49.3%) became federally authorized patients in 2004 or later, while 50.7% joined the program prior to 2004. When asked if they had difficulty finding a physician to support their application, exactly 50% said "yes", and 50% answered "no", reflecting the diversity and unpredictability of medical support available throughout Canada. In terms of processing applications, 35.3% had theirs completed by Health Canada within 2-4 months, and 29.4% state that it took 60 days or less. However, 35.2% of participants suggest that it took over 4 months, with 17.6% citing that they waited over 12 months for their application to be processed. This suggests that for those suffering from serious or terminal conditions, processing times would be a significant concern and may not be quick enough to allow some patients to legally use cannabis in end-of-life situations.

The following set of 6 questions put three statements with positive connotations and 3 statements with negative connotations to survey respondents, and are based on standardized and validated Short-Form Patient Satisfaction Questionnaire (PSQ-18) traditionally used to evaluate health service delivery at hospitals, clinical and insurance companies. In addressing the statement "I find the application for a federal authorization simple and uncomplicated", only 21.8% "agreed" or "strongly agreed", while 71.2% "disagreed" or "strongly disagreed" (42.5%), suggesting that for most patients the federal application process is onerous and challenging. When asked to comment on the statement "Employees at Health Canada's MMAD act too businesslike and impersonal towards me", 54% "agreed" or "strongly agreed", while 28.7% "disagreed" or "strongly disagreed". In regards to the statement "I am dissatisfied with the service I receive from Health Canada in regards to my use of medical cannabis", 68.9% "agree" or "strongly agree", while only 18.3% "disagree" or "strongly disagree". However, when asked if "Employees at Health Canada's MMAD treat me in a friendly and courteous manner", respondents were split, with 35.6% "agreeing" or "strongly agreeing", 27.6% "uncertain", and 36.8% "disagreeing" or "strongly disagreeing". When the statement "I have full confidence in the ability of the Health Canada employees that administer this program" was put to patients, 76.8% "disagreed" or "strongly disagreed", with only 5.9% "agreeing" or "strongly agreeing" with the statement, and 17.4% stating that they were "uncertain". Finally, when asked "I am able to get help from Health Canada in regards to my medical use of cannabis whenever I need it", 8.2% "agreed" or "strongly agreed", while 70.6% "disagreed" or "strongly disagreed", with 21.2% uncertain.

**Figure 5:**
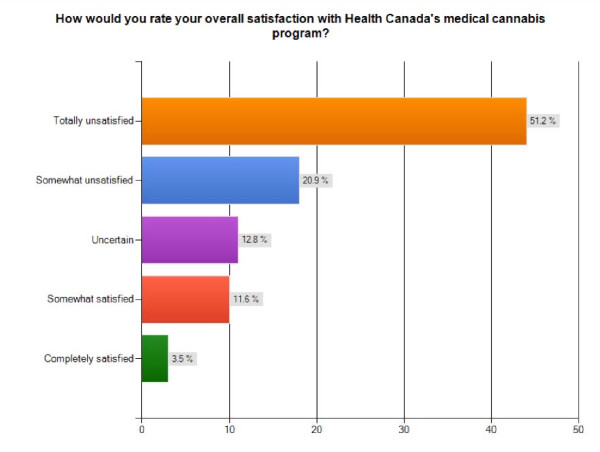
**Overall Satisfaction with Health Canada Medical Cannabis Program**. Bar graph of overall level of satisfaction with Health Canada's medical cannabis program reported by survey participants (n = 86).

The final question of the survey asked participants to rate their overall satisfaction with Health Canada's medical cannabis program, and 15.1% of patients state that they are "completely" or "somewhat satisfied", 12.8% uncertain, and 72.1% either "somewhat" (20.9%) or "totally unsatisfied" (51.2%) (Figure 5). This suggests a very poor patient perception of the service quality at Health Canada Marihuana Medical Access Division, with many potential improvements in application processing times, cannabis selection and quality and overall responsiveness to patient queries and concerns.

In a federally-funded report titled "Our Rights, Our Choice,' which examined the human rights, ethical and legal challenges faced by people living with HIV/AIDS who choose to use medical cannabis, the Canadian AIDS Society found that although between 14 to 37% of people living with HIV/AIDS used cannabis to address their condition, many had faced hurdles accessing the federal program. The CAS report states that:

access to the federal program remains hindered by barriers such as a lack of awareness of the program's existence, mistrust in the government, misinformation about the program and difficulty in finding a physician to support their application. Thousands of seriously ill Canadians must therefore choose between breaking the law to use the therapy of their choice, or going without, which in many cases compromises their well-being and quality of life [6].

The results of this federally authorized medical cannabis patient survey support the findings of the CAS study and other research into the MMAR/MMAD [3-5].

## Discussion

Creating policies and procedures for safe patient access to medical cannabis has proven to be a challenge in Canada and around the world. In the U.S., the 14 states that allow for the legal use of cannabis continue to struggle to protect patients, address access issues, and mitigate community concerns, all of which is made all the more complicated by ongoing resistance and active legal threats by the federal government [7]. In Canada, patients face multiple challenges to safe access: 1) resistance from the medical community to act as gatekeepers to the program; 2) an onerous application process; 3) a very limited and much-criticized cannabis supply; 4) limited income and a lack of national cost-coverage; and 5) ongoing social prejudice against the use of medical cannabis [3-6]. Results from this survey suggest that reducing bureaucratic obstacles while increasing patient options for access would result in greater levels of patient participation and overall satisfaction with the federal program.

While there is a remarkable diversity in the demographics and medical conditions of cannabis patients, some common themes emerge from this research. It is clear that patients' would like to have a choice of many different strains and forms of ingestion in order to more safely and effectively address their many different symptoms and conditions. Since cost continues to be a significant obstacle for patients with low or fixed income, provincial or federal cost-reduction or coverage policies should be implemented [5,6]. The high bureaucratic burden on both patients and physicians is reducing participation in the program, so allowing healthcare providers to treat cannabis like any other medicine would likely improve uptake and might also alleviate some of the social stigma associated with the therapeutic use of cannabis. Since this study and Health Canada's own statistics [21] show that the majority of participants in the Canadian federal program chose to produce their own medicine, policies and procedures should be put in place that maintain the option of personal production while also ensuring that both patients and communities are protected from the dangers of poorly-cultivated cannabis. This could range from basic information from Health Canada on safe production practices to electrical inspections at the municipal level. Additionally, with over half of respondents currently accessing cannabis through dispensaries and growing evidence that these organizations build social capital and provide an environment that is conducive to health and healing [3-7], the federal government should work with dispensaries to develop regulations that would incorporate this community-based model of access into Canada's medical cannabis program.

Finally, many of the challenges faced by the MMAD could have been addressed or avoided through a more robust and active strategy for patient engagement and involvement. Although there are many stakeholders directly or indirectly affected by the federal medical cannabis program - municipalities, police, physicians, etc. - the key stakeholders are the Canada's critically or chronically ill who could or do benefit from the use of cannabis. Unfortunately, the short history of the MMAR/MMAD shows that the needs and concerns of patients has all too often been ignored or overshadowed by other interests and concerns [3-6]. The future success of this cutting-edge program will depend largely on the willingness of the federal government to create a truly patient-centered approach to medical cannabis access, including active and ongoing engagement with end-users, support for research into the potential harms and benefits of medical cannabis, and increased options for patients, potentially through the regulation of community-based dispensaries.

There are a few limitations to this study. Although participants represented about 5% of the patient population in the program at the time of the survey there is no way to know how representational this cohort is to the rest of the participants in the MMAD since Health Canada has never released any demographic information about federally authorized users. Additionally, since recruiting was largely done online and through medical cannabis patient lists and groups, it is possible that this more active population has a higher level of dissatisfaction with the federal program. However, the general demographics of participants in this study is similar to those identified by Reinarman in a recent U.S.-based study [19], and many of the patient needs and challenges that came to light in this survey support previous research on Canada's medical cannabis population and associated federal program [3-6]. It is hoped that this survey, which represents the first polling ever conducted solely on federally authorized patients in Canada, will assist policy-makers here and abroad develop more patient-centered strategies for safe access to medical cannabis.

## Competing interests

Philippe Lucas is the founder of the Vancouver Island Compassion Society (VICS), and was employed as Executive Director of during the design and data gathering portions of this study. While he is no longer an employee of the VICS, he remains a member of the board.
